# Unique cellular interactions between pancreatic cancer cells and the omentum

**DOI:** 10.1371/journal.pone.0179862

**Published:** 2017-06-20

**Authors:** Valerya Feygenzon, Shelly Loewenstein, Nir Lubezky, Metsada Pasmanic-Chor, Osnat Sher, Joseph M. Klausner, Guy Lahat

**Affiliations:** 1 Sackler School of Medicine, The Nicholas and Elizabeth Cathedra of Experimental Surgery, Tel Aviv University, Tel Aviv, Israel; 2 Department of Surgery, Tel Aviv Sourasky Medical Center, Tel Aviv, Israel; 3 Bioinformatics Unit, Tel Aviv University, Tel Aviv, Israel; 4 Department of Pathology, Tel Aviv Sourasky Medical Center, Tel Aviv, Israel; University of South Alabama Mitchell Cancer Institute, UNITED STATES

## Abstract

Pancreatic cancer is a common cause of cancer-related mortality. Omental spread is frequent and usually represents an ominous event, leading to patient death. Omental metastasis has been studied in ovarian cancer, but data on its role in pancreatic cancer are relatively scarce and the molecular biology of this process has yet to be explored. We prepared tissue explants from human omental fat, and used conditioned medium from the explants for various in vitro and in vivo experiments designed to evaluate pancreatic cancer development, growth, and survival. Mass spectrometry identified the fat secretome, and mRNA array identified specific fat-induced molecular alternations in tumor cells. Omental fat increased pancreatic cancer cellular growth, migration, invasion, and chemoresistance. We identified diverse potential molecules secreted by the omentum, which are associated with various pro-tumorigenic biological processes. Our mRNA array identified specific omental-induced molecular alternations that are associated with cancer progression and metastasis. Omental fat increased the expression of transcription factors, mRNA of extracellular matrix proteins, and adhesion molecules. In support with our in vitro data, in vivo experiments demonstrated an increased pancreatic cancer tumor growth rate of PANC-1 cells co-cultured for 24 hours with human omental fat conditioned medium. Our results provide novel data on the role of omental tissue in omental metastases of pancreatic cancer. They imply that omental fat secreted factors induce cellular reprogramming of pancreatic cancer cells, resulting in increased tumor aggressiveness. Understanding the mechanisms of omental metastases may enable us to discover new potential targets for therapy.

## Introduction

Pancreatic ductal adenocarcinoma (PDAC) ranks fourth in cancer- related mortality in the U.S., eighth worldwide, and is among the most devastating of human malignancies [1, 2]. It is characterized by high rates of local invasion, distant metastasis and resistance to chemotherapy and radiation. To date, surgical resection is the only potentially curative therapeutic option; however, most patients are inoperable at the time of diagnosis due to metastatic disease [3, 4]. Pancreatic cancer, like other gastrointestinal (GI) malignancies, usually metastasizes to the liver and the peritoneal cavity, where omental involvement is not uncommon [5, 6]. Present conventional systemic therapy is ineffective for the treatment of PDAC peritoneal metastasis; therefore, omental spread which is characterized by aggressive tumor growth, rapid patient deterioration and inevitable mortality is considered an ominous event in the course of the disease.

Most of the omentum is composed of adipose tissue bands that contain adipocytes, blood and lymph vessels, immune cells and stromal cells. All these omental cellular components constitute the immediate microenvironment of metastatic cells, forming a perfect soil for their seeding, survival and proliferation. Although knowledge concerning the role of fibroblasts, endothelial cells, and even adipocytes in cancer progression is expanding [7–9], data pertaining to the molecular mechanisms related to the process of omental metastasis is scarce, and mostly relate to ovarian cancer [10–12]. To the best of our knowledge, experimental data concerning PDAC omental metastasis is limited.

There is a critical need for novel treatment strategies targeting PDAC omental metastasis, and their development requires a better understanding of the molecular basis of omental spread. Towards this aim, we sought to investigate the potential interactions between the omentum and PDAC cells in order to delineate the active role of the omental fat in the progression of PDAC omental metastasis, and to identify potential genes related to this process.

## Materials and methods

### Cell culture

PANC-1 and MIA-PaCa-2 human pancreatic adenocarcinoma cells were purchased from the American Tissue Culture Collection (ATCC). Both types of cells were cultured in Dulbecco’s modified Eagle’s medium (DMEM) supplemented with 10% fetal calf serum (FCS) and 100 U/ml penicillin-streptomycin (Biological Industries, Beit Haemek, Israel). The cells were maintained in a humidified 5% CO_2_ atmosphere at 37°C.

### Human sample collection and conditioned medium (CM) preparation

The study protocol was approved by the Human Ethics Review Committee of the Israeli Ministry of Health and the Tel Aviv Sourasky Medical Center. A written informed consent was obtained for each of the participating patients. Fresh human omental fat was harvested from pancreatic cancer surgical patients. Inclusion criteria were operable pancreatic cancer, no evidence of peritoneal spread during surgery, no evidence of parenchymal involvement according to preoperative CT scan and BMI< 30. All patients were lacking a metastatic disease; however, nodal status, the presence of lymphovascular invasion (LVI) and level of differentiation differed. The omental fat tissues were harvested from approximately 50 patients undergoing pancreatic cancer surgery. Each experiment was repeated at least three times, utilizing a different sample for every experiment.

Adipose tissue explants were prepared as described elsewhere [13]. Briefly, cultured omental tissue fragments (2–3 mm^3^, 100 mg/ml medium) were incubated at 37°C in medium [M199 (Invitrogen) + 10% (v/v) FBS, 2 mM L-glutamine] and allowed to settle for 2 hours. The medium was replaced, and the fragments were further incubated for 24 hours in serum-free M199 (0.5% BSA). Under these conditions, the explants remain viable and functional for at least 48 hours. The viability of adipose tissue explants after 24 hours of incubation was also documented by the activity of lactate dehydrogenase (LDH) in the medium after 24 hours. LDH activity in adipose tissue lysate was used as the positive control. In all experiments, LDH values for all samples were within normal limits for control cells (12.6%±4.9%). Fragments were removed with tweezers, and the CM was transferred from the well to a clean tube and immediately utilized or quickly frozen (10 seconds) in liquid nitrogen and stored at -80°C. For control, we used the medium in which omental fat cells explants were prepared and incubated (M199 regular medium-RM) prior to the collection of the CM. For the mass spectrometry analysis, CM was collected after 24 hours in M199 medium without serum.

### Cell growth assay

Cell proliferation was measured using the XTT cell proliferation kit (Biological Industries, Israel) according to the manufacturer's instructions. Briefly, 5000 cells/well were plated in a 96-well plate and incubated with RM or CM for 24 hours. Absorbance was measured at a wavelength of 490 nm, and relative cell growth was expressed as fold change relative to control RM-treated cells. For cell inhibition assay, the cells were incubated with RM or CM and increasing doses of gemcitabine (0.05–50 μM) [14, 15]. For anchorage independent growth, 5000 viable PANC-1 or MIA-PaCa-2 cells were plated in a 12-well plate in RM or CM containing 0.3% low-melting agarose overlying a 0.5% agarose layer in RM or CM, respectively. The cells were incubated for 21 days at 37°C, and the formed colonies were analyzed morphologically by quantifying the number of colonies per well. All experiments were repeated three times for each cell line with CM from three different patients.

### Migration and invasion assays

Transwell migration and invasion assays were conducted as described elsewhere [16]. Briefly, 4x10^4^ PANC-1 and MIA-PaCa-2 cells incubated with CM or RM for 24 hours were placed in the upper chamber and allowed to migrate into 24-well transwell inserts with 8 μm pores (BD Biosciences). The lower chamber was filled with 0.75 ml/well of RM supplemented with 1% fetal bovine serum. Invasion assays were conducted similarly using 24-well transwell inserts with 8 μm pore polycarbonate filters coated with Matrigel (BD Biosciences). For invasion only, cells suspended in RM (0.5ml/camber) were placed in the upper chamber and allowed to invade into 24-well transwell inserts with 8 μm pore size coated with Matrigel while the lower chamber was filled with 0.75 ml/well of RM or CM supplemented with 1% FBS. In all cases, the cells were incubated for 16 hours at 37°C. After incubation, the filters were fixed with 4% formaldehyde and stained with 0.2% crystal violet. Cells on the upper surface of the filters were removed by wiping with a cotton swab, and migratory and invasive activities were determined by counting the number of cells in three fields per well (magnification, X100) in triplicates. The number of migrated or invaded cells was quantified by counting cells with the ImageJ 1.48v.Java image processing program.

### Scratch wound healing assay

PANC-1 and MIA-PaCa-2 cells were seeded in a 6-well tissue culture plate and allowed to grow to ~95% confluence. The plates were scratched with a 200 μl pipette tip across the center of the well to create a straight line. Another straight line was scratched perpendicular to the first line to create a cross in each well. The cells were washed twice with phosphate buffered solution (PBS) to remove any detached cells, and fresh RM or CM was added to each well. Images were captured under the microscope at 0 and 24 hours to assess the rate of gap closure.

### Apoptosis assay

Apoptosis was measured using an apoptosis kit (MEBCYTO Apoptosis Kit, MBL) according to the manufacturer’s recommendations. In brief, 1x10^6^ PANC-1 and MIA-PaCa-2 cells were incubated with omental fat CM or RM and increasing doses of gemcitabine (0.05–10 μM) [14, 15] for 24 hours. The cells were then trypsinized, washed and resuspended in binding buffer. After the addition of annexin V-FITC and propidium iodide (PI) to the cell suspension, the cells were incubated for 15 minutes in the dark and measured by FACS (BD FACS Canto II) using a single laser emitting excitation light at 488 nm.

### Cell cycle analysis

1x10^6^ cells were incubated with CM or RM for 24 hours, trypsinized, centrifuged and resuspended with 300 μl of cold PBS. The cells were fixed with 70% cold ethanol, treated with 5 μg of ribonuclease A and 10 μg of propidium iodide (PI), after which they were analyzed by FACS.

### Liquid chromatography and tandem mass spectrometry (LC-MS/MS)

CM from explants of human visceral fat and subcutaneous (SC) fat (*n* = 6) were collected as described elsewhere [17]. Each sample was subjected to buffer exchange with Amicon 3 kDa molecular weight filters (Millipore) into 50 mM ammonium bicarbonate. Protein concentration from each sample was determined using a BCA assay. The samples were then subjected to in-solution tryptic digestion. Proteins were first reduced by incubation with 5 mM dithiothreitol (Sigma) for 30 minutes at 60°C and alkylated with 10 mM iodoacetamide (Sigma) in the dark for 30 minutes at 21°C. Trypsin (Promega; Madison, WI, USA) was then added at a 1:50 tryptisn:protein ratio for 16 hours at 37°C. The digestions were stopped by trifluroacetic acid (1%). The samples were desalted using solid-phase extraction columns (Oasis HLB, Waters, Milford, MA, USA) and stored at -80°C until further analysis. The samples were fractionated using high pH reversed phase, as described in (http://genome.cshlp.org/content/22/7/1231.short/). The fractions were then analyzed using nanoflow chromatography coupled with high-resolution mass spectrometry, as described in (http://pubs.acs.org/doi/abs/10.1021/pr501045t).

Raw data were imported into a Rosetta Elucidator System, version 3.3 (Rosetta Biosoftware, Seattle, WA, USA). The Elucidator was used for alignment of raw MS1 data in retention time (RT) and mass charge (m/z) dimensions as described elsewhere [18]. The MS/MS spectra generated by the Elucidator were sent for a sequence database search using Mascot version 2.4. The data were searched against the forward and reverse human sequence in UniprotKB version 2013_04 appended with 125 common laboratory contaminants. A variable modification was set to oxidation of M and a fixed modification was set to carbamidomethylation of C. MS tolerance was set to 10 ppm and MS/MS tolerance to 0.02 Da. The resultant identifications were then imported back to the Elucidator and filtered for a maximum 1% false discovery rate at the protein level. Relative protein abundance was calculated using the Hi-3 method [19].

### In vivo animal models

All animal procedures and care were approved by the Sourasky Institutional Animal Care and Usage Committee (Permit Number: 2n-1-15). Animals received humane care as per the Animal Welfare Act and the NIH “Guide for the Care and Use of Laboratory Animals”. PANC-1 (1x10^6^/0.1 mL PBS/mouse) cells were pre-incubated in vitro for two days with human omental fat CM or non-conditioned medium prior to their subcutaneous (SC) injection into the flank of six-week-old male Athymic nude mice Foxn1^nu/+^ (n = 5/experiment). Mice were followed for tumor size, wellbeing, and body weight and sacrificed when any group tumors reached an average of 1.5 cm in its largest dimension. Tumors were resected, weighed, and frozen or fixed in formalin and paraffin-embedded for H&E staining. Tumor volume was calculated using the following formula: tumor volume = LxW^2^xπ/6 (cm^3^) where L is the tumor’s length and W is its width.

### Immunohistochemistry

Immunohistochemistry (IHC) was performed with the Ventana Benchmark automated staining system (Ventana Medical Systems, Tucson, AZ) on 4-μm paraffin sections. The slides were deparaffinized in xylene and rehydrated through a graded series of ethanol concentrations. Tissue sections were stained using the following primary antibody: Ki67 (1:100, Spring Bioscience, California USA); CD31 (1:100, Cell Marque, California USA); S100 (1:100, Cell Marque, California USA) and loaded into a Benchmark XT (Ventana Medical Systems Inc, Tucson, AZ) automated stainer. Primary antibodies were detected with the Ventana iVIEW DAB detection kit. Scoring of Ki67 and CD31 protein expression was interpreted independently by an expert gastro-intestinal pathologist (SO). For quantification of the proliferation, the percentage of Ki67-positive nuclei was determined in five of the most proliferating areas within a tumor (‘‘hot spots”) (x200 magnification, *n* = 10). To quantify angiogenesis, blood vessels/cells were counted in a representative high-power (x200) field. Blood vessel density was calculated as the mean ±SD of all counts (x200 magnification, *n* = 10).

### RNA microarray

Total RNA was extracted from PANC-1 cells treated with RM (*n* = 3) or CM (*n* = 9) using Tri Reagent (Sigma, Life Sciences). Total RNA concentrations were measured using a Nanodrop ND-1000 spectrophotometer (NanoDrop Technologies, Wilmington, DE). The cDNA was hybridized to Affymetrix^®^ Human Gene 2.1 ST Array Strip (Affymetrix, Santa Clara, CA). Hybridized chips were stained, washed, and scanned with the Affymetrix GeneChip 3000 7G plus scanner (Affymetrix). Microarray analysis was performed using Partek Genomics Suite version 6.6 (Partek, St. Louis, MO). Data were normalized and summarized with the robust multiaverage method [20]. Heat maps were generated using Partek Genomics Suite software with Pearson’s dissimilarity correlation and average linkage methods. Functional enrichment analysis was performed using DAVID and GOEAST tools.

### Quantitative real-time PCR analysis

Total RNA was isolated from the cells using TRizol reagent (Invitrogen). One microgram of total RNA was reverse-transcribed to cDNA using the random hexamers method. Quantitative real-time PCR reactions were performed in triplicate with SYBR Green PCR mix according to manufacturer’s instructions (Applied Biosystems) in an Applied Biosystems StepOnePlus Real-Time PCR System using appropriate primer sets (Sigma). The calculation of relative change in mRNA was standardized to the housekeeping gene GAPDH.

### Western blot analysis

Total protein extracts were prepared in ice-cold RIPA lysis buffer with 1% protease inhibitor (Sigma-Aldrich). Cell lysates were then centrifuged at 12000 rpm for 10 minutes at 4°C and the supernatants were collected. The total protein concentration in the cell lysates was determined using the Bio-Rad Protein Assay reagent (Bio-Rad, Hercules, CA, USA). Equal amounts of protein were separated via SDS-PAGE and transferred to nitrocellulose membranes. The membranes were blocked in Phosphate-Buffered Saline with Tween-20 (PBST) that contained 5% skim milk and incubated with antibodies against OPN (1:100 dilution, Santa Cruz Biotechnology) and α-actinin (1:500 dilution, Santa Cruz Biotechnology) overnight at 4°C. The membrane was then washed with Phosphate-Buffered Saline with Tween-20 (TBST) three times and incubated with secondary goat anti-mouse antibody for 90 minutes at room temperature. Protein bands were visualized by enhanced chemiluminescence (Pierce, Rockford, IL).

### Statistical analyses

Statistical analysis was performed using GraphPad Prism^TM^ software. Numerical values of cell culture and mouse xenograft data were analyzed using Student's t-test for significance. Results are expressed as means ± SD. All figures are representative results of at least 3 independent experiments. The indicated number of experiments (n) refers to the experiments performed with omentum from different patients. A *P* value ≤0.05 was considered as being significant.

## Results

### Omental fat induces pancreatic cancer cell growth

**Fig 1:**
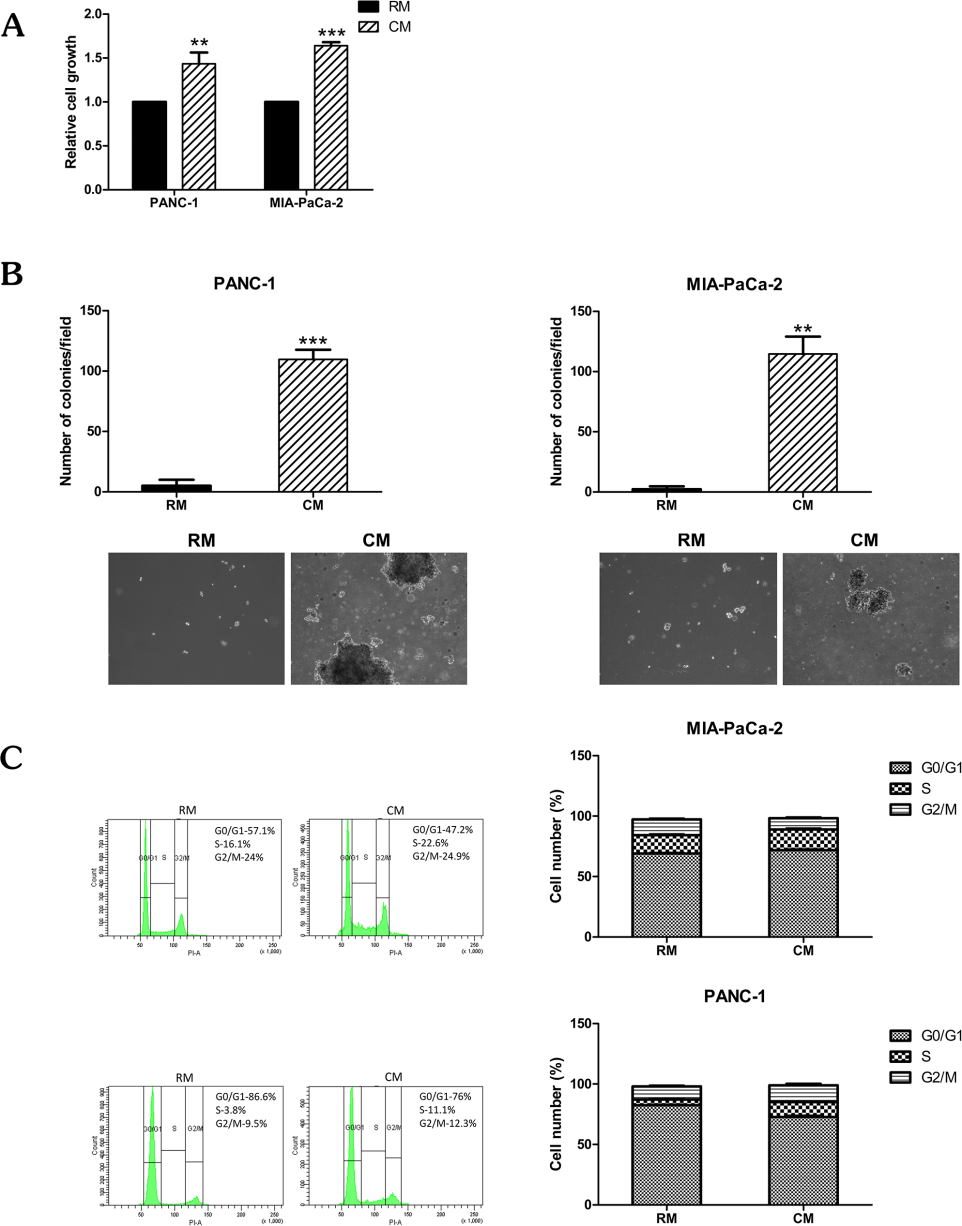
Omental fat CM enhances pancreatic cancer cell growth. (A) XTT assay demonstrating a significant increase (*P*< .01) in proliferation of pancreatic cancer cells after incubation with omental fat CM, n = 7; (B) Omental fat CM markedly increased pancreatic cancer cell colony formation capacity (*P*< .01). The upper panel graphs represent the average of four repeated independent experiments ±SD, and the lower panel depicts representative images of cell colonies in soft agar (magnification, X100); (C) Omental fat CM-induced S-phase population in pancreatic cancer cells; a more pronounced effect was seen in PANC-1 cells than in MIA-PaCa-2 cells (*P*< .05). Bar plots display the data of 7 independent experiments.

We used two human pancreatic cancer cell lines, PANC-1 and MIA-PaCa-2, to evaluate the potential effects of human omental fat CM on pancreatic cancer cellular proliferation and cell cycle. The cells were incubated with CM or RM, and the XTT proliferation assay demonstrated that omental fat CM significantly increased cellular proliferation of both pancreatic cancer cell lines in comparison to RM (*P*< .01; Fig 1A). In addition, we investigated the effect of omental fat CM on anchorage-independent growth of both cell lines: the capacity of those cells to grow in soft agar was significantly increased after incubation with omental fat CM (*P*< .01; Fig 1B). Based on the observed effect of omental fat CM on pancreatic cancer cell growth, we next evaluated its effect on cell cycle progression. Following treatment of the PANC-1 cells with omental fat CM, the cell cycle analysis demonstrated a significant increase of approximately 150% in the S-phase population compared to RM (*P*< .001; Fig 1C). A smaller but still significant effect was observed in MIA-PaCa-2 cells (*P*< .05; Fig 1C). Taken together, these data suggest that omental fat CM promotes pancreatic cancer cell growth and proliferation. This may be explained, at least in part, by fat-induced cell cycle deregulations.

### Omental fat enhances pancreatic cancer cell migration and invasion

**Fig 2:**
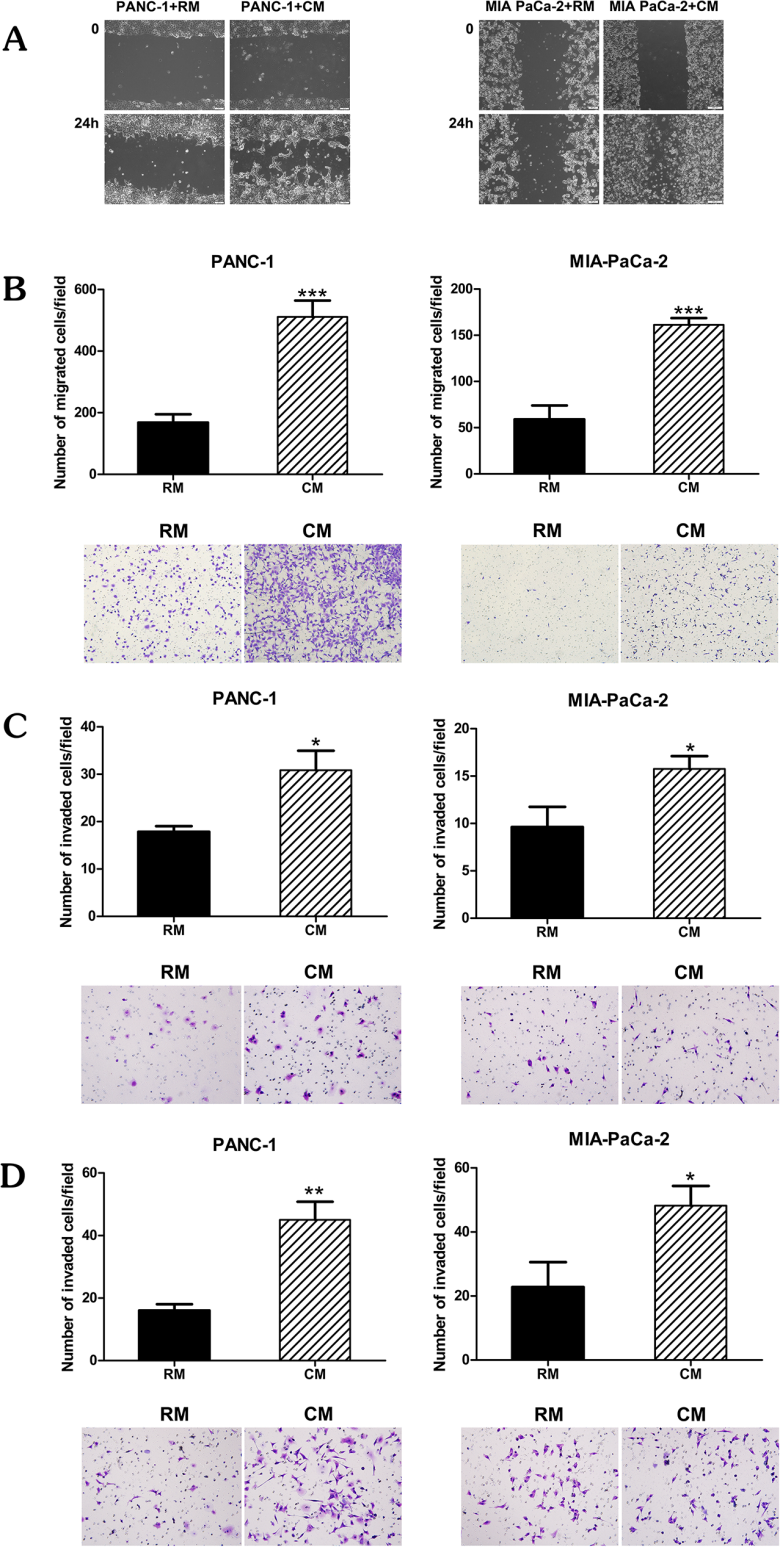
Omental fat CM enhances pancreatic cancer cell migration and invasion. (A) Wound healing scratch assay demonstrating the effect of omental fat CM on PANC-1 and MIA-PaCa-2 cell migration; Scale bar = 200 μm. (B); Modified Boyden chamber assays depicting the effects of omental fat CM on pancreatic cancer cell migration (*P<* .001); (C) Matrigel invasion chamber demonstrating a significant increase in invasion of PANC-1 and MIA-PaCa-2 cells pre-treated with omental fat CM (*P*< .05); (D) Increased invasion of PANC-1 and MIA-PaCa-2 cells by using omental fat CM as a chemoattractant (*P* < .01). The upper panel graphs represent the average of 5 repeated experiments ± SD, and the lower panel depicts representative images (magnification, X100).

We next evaluated the effect of omental fat CM on pancreatic cancer cell migration and invasion. Pancreatic cancer cells were pretreated with CM or RM for 24 hours, at which point a scratch wound healing assay was conducted. We observed a marked increase in migration (Fig 2A). In addition, modified Boyden chambers were used to quantitate the effect of omental fat CM on migration and invasion. The pancreatic cancer cells were pretreated with CM or RM for 24 hours, washed and counted, and only the viable cells were further utilized. Omental fat CM significantly increased PANC-1 and MIA-PaCa-2 cell migration by >3-fold and 2.5-fold increase, respectively (*P*< 0.001; Fig 2B). As depicted in Fig 2C, omental fat CM also enhanced PANC-1 and MIA-PaCa2 invasion, > 2-fold and >1.5-fold, respectively (*P*< .05; Fig 2C). We next evaluated whether omental fat CM harbors chemotactic properties. Non- treated pancreatic cancer cells were incubated for 24 hours in the upper chamber while the lower chambers contained omental fat CM or RM. The omental fat CM significantly increased the number of cells invading through the matrigel towards the omental fat CM, with a >2-fold increase for both cell lines (*P*< .05; Fig 2D). These results demonstrate that omental fat CM-secreted factors increase pancreatic cancer cell motility and invasiveness; it is possible that some of these factors play a role in chemotaxis.

### Omental fat augments pancreatic cancer cell chemoresistance

**Fig 3:**
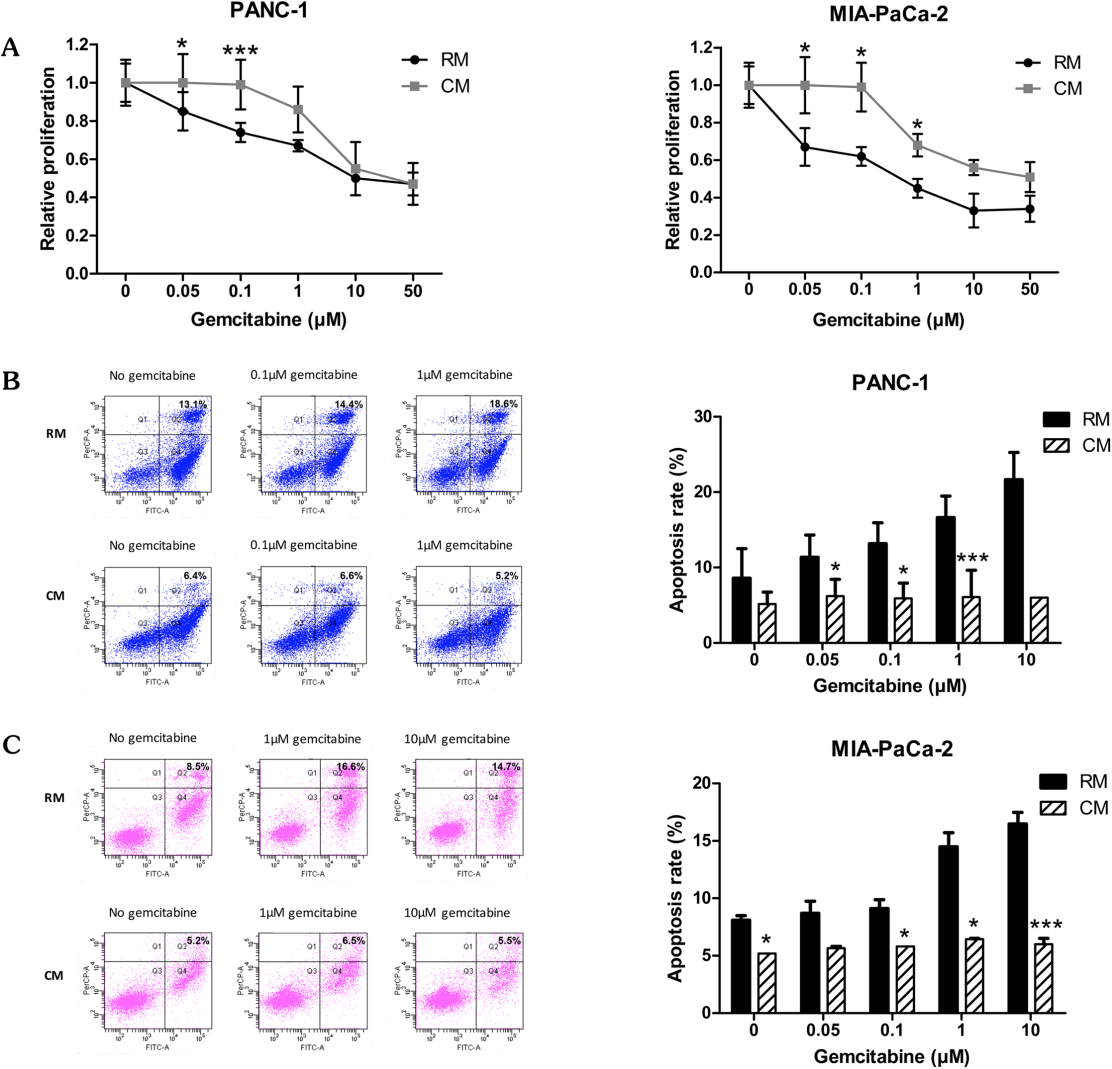
Omental fat CM augments pancreatic cancer cell chemoresistance. (A) XTT assay demonstrating a significant increase in survival of gemcitabine-treated pancreatic cancer cells following incubation with omental CM (*P*< .05), n = 4; (B and C) Annexin-V/PI FACS analysis demonstrating a marked reduction in gemcitabine-induced apoptosis of PANC-1 (B; *P*< .05) and MIA-PaCa-2 cells (C; *P*< .05) pretreated with omental fat CM, n = 5.

Evasion of apoptosis is one of several distinct features in the process of tumorigenesis [21]. We hypothesized that pancreatic cancer cells pretreated with omental fat CM would demonstrate resistance to gemcitabine, the conventional anti-pancreatic cancer chemotherapeutic agent [22]. To test this hypothesis, PANC-1 and MIA-PaCa-2 cells pretreated with omental fat CM or control medium were exposed to increasing doses of gemcitabine (0.05–50 μM), and chemosensitivity was initially determined by XTT assay. PANC-1 and MIA-PaCa-2 cells pretreated with omental fat CM demonstrated a significantly higher survival rate after gemcitabine treatment compared with control medium, specifically, >1.3-fold for 0.1μM gemcitabine in PANC-1 and >1.5-fold for 0.1 μM gemcitabine in MIA-PaCa-2 cells (*P*< .05; Fig 3A). To further evaluate the plausible chemoprotective effect of omental fat CM on pancreatic cancer tumor cells, we conducted FACS analyses using Annexin V-FITC and PI staining. PANC-1 and MIA-PaCa-2 cells were treated with increasing doses of gemcitabine for 24 hours. Annexin V and PI staining demonstrated a significant decrease in the number of apoptotic events in both pancreatic cancer cell lines that were cultured in omental fat CM compared with RM, specifically, 5.2% versus 18.6% (*P*< .001) in the PANC-1 cells (Fig 3B) and 5.5% versus 14.7% (*P*< .001) in the MIA-PaCa-2 cells (Fig 3C). Taken together, these data imply that omental fat CM has a pro-tumorigenic effect on pancreatic cancer tumor cells, including enhancement of tumoral growth and survival as well as increased cellular motility and invasiveness. Moreover, our results indicate that it is possible that omental fat has an active role in the overt resistance to chemotherapy which is frequently observed among patients with omental spread of pancreatic cancer.

### Human omental fat promotes pancreatic cancer xenograft growth in nude mice

**Fig 4:**
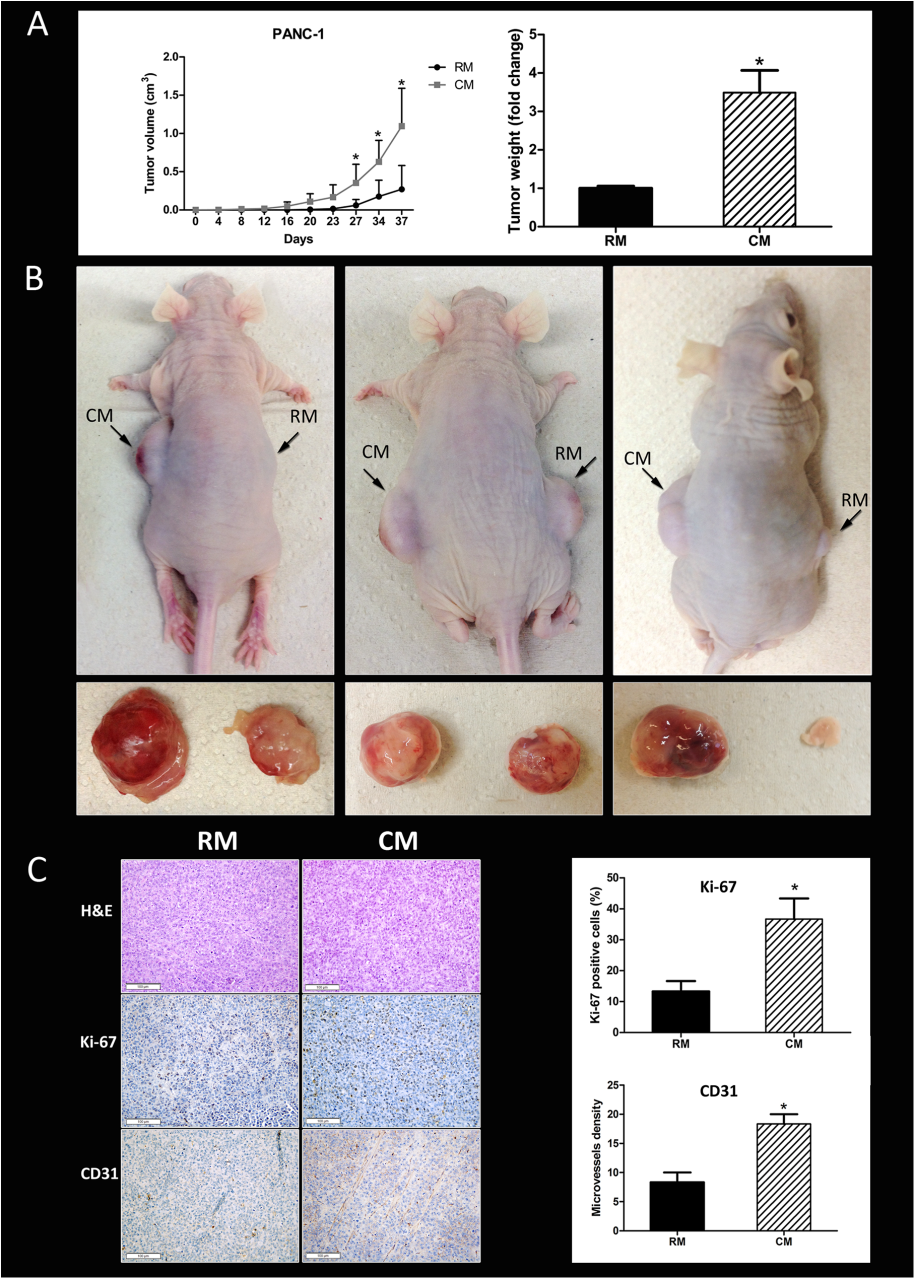
Tumor growth is promoted by omental fat in vivo. (A) Facilitation of tumor growth and weight of PANC-1 tumors in mice following pre-treatment with omental fat CM (n = 15). Graphs represent the average of three repeated experiments ±SD (*P<* .*05*); (B) Representative tumor and mice images. (C) Marked increase in proliferation (Ki-67) and microvessel density (CD31) by human omental fat CM. Representative immunohistochemistry (IHC) images are shown on the left (H&E, x200; Ki-67, x200; CD31, x200). IHC quantification is shown on the right. n = 10 in tissues of each site; Scale bar = 100 μm.

Thus far, our data suggested that omental fat CM increases pancreatic cancer cellular aggressiveness, an effect probably mediated by factors secreted by the diverse cellular components of the omentum. Next, we sought to evaluate whether these mediators augment pancreatic cancer tumor growth in vivo. For that purpose, we used a heterotopic human pancreatic cancer model in nude mice. The PANC-1 pancreatic cancer cells were initially pretreated in vitro with human omental fat CM or control RM. The tumor cells were then injected subcutaneously into the flank of nude mice. PANC-1 cells pretreated with omental fat CM grew faster than the control tumors growing in the contralateral flank: the xenograft tumor volumes were 1.09±0.4 cm^3^ versus 0.27±0.19 cm^3^, respectively (*P*< .05; Fig 4A). The mean tumor weight of omental fat CM-treated cells was almost 4-fold higher than tumors produced by PANC-1 cells pretreated with control non-conditioned RM, 0.65±0.1 gr vs. 0.19±0.04 gr, respectively (*P*< .05; Fig 4A). Fig 4B depicts representative mice and tumors harvested from both flanks. Based on our in vitro data, we performed IHC staining for Ki67 and CD31 for proliferation and angiogenesis, respectively. PDAC cellular proliferation in vivo was significantly induced by the omental fat (Fig 4C). The Ki-67 scores in tumors pretreated with omental fat CM and in tumors pretreated with RM were 36.66±3.85 vs. 13.33±1.92 percent, respectively (*P*< .05). In addition, CD31 IHC staining demonstrated a significantly increased microvessel density in tumors pretreated with CM compared with those pretreated with RM, 20±2.02 vs. 8.33±0.9, respectively (*P* < .05; Fig 4C).

### The omental fat secretome includes various proteins related to cancer progression and metastasis

Visceral fat and the omentum in particular consist of a stromal vascular fraction which includes various cells and adipocytes. Since all of these cells contribute to the secretome and its metabolic effects, we sought to characterize omental fat as a whole rather than the isolated adipocytic secretome. For that purpose, we used Liquid Chromatography and Tandem Mass Spectrometry (LC-MS/MS) on CM from human omental versus SC fat explants. The identified proteins were analyzed with the Ingenuity Pathways Analysis (IPA) according to their distinct cellular location: cytoplasm, nucleus, plasma membrane, and extracellular space. Of the 800 proteins identified in the LC-MS/MS analysis, 194 are extracellular. We further analyzed these 194 proteins according to their molecular and cellular function: 88 are related to cellular movement, 102 to cellular growth and proliferation, 94 to cell death and survival, and 94 are involved in cell-to-cell signaling and interaction. IPA analysis identified 157 proteins that are related to cancer development, progression and/ or metastasis. Moreover, we specifically searched for previously reported pancreatic cancer- related molecules. That analysis explored a list of nine relevant proteins (NGAL, FINC, ZA2G, PGS1, TIMP1, MSLN, IL-6, MMP8 and TSP1; Table 1), of which seven were up-regulated and two were down-regulated (*P*< .05). They all had been reported as pro-tumorigenic proteins in pancreatic cancer, affecting tumor progression and/or spread [23–29]. IPA also identified potential upstream regulators, which play a key role in cancer invasiveness: interleukin-6 (IL-6), IL1B and transforming growth factor, beta-induced (TGFBI) were the top three upstream activated regulators (*P*< .05).

**Table 1:** Cancer-related proteins identified by LC-MS/MS analysis of human omental fat (OF) versus subcutaneous fat (SC).

Protein Name	Protein Description	Fold Change (OF/SC)
MSLN	Isoform 3 of mesothelin	20.55
TIMP1	Metalloproteinase inhibitor-	12.71
MMP8	Matrix metalloproteinase-8	9.10
IL-6	Interleukin-6	6.00
H9KV70	Neutrophil gelatinase-associated lipocalin	5.36
FINC	Fibronectin	3.01
TSP1	Thrombospondin-1	1.54
PGS1	Biglycan	-2.03
ZA2G	Zinc-alpha-2-glycoprotein 1	-1.31

These results provide solid evidence that the omentum secretes various cancer-related proteins which contribute to cellular proliferation, cell survival, and angiogenesis, thus, strengthening the results we obtained in our in vitro and in vivo experiments. Nevertheless, further research is needed in order to characterize specific pathways involved in the potential crosstalk between the omentum and the metastatic pancreatic cancer cells.

#### Gene expression profiles of PANC-1 cells treated with omental fat conditioned medium

**Fig 5:**
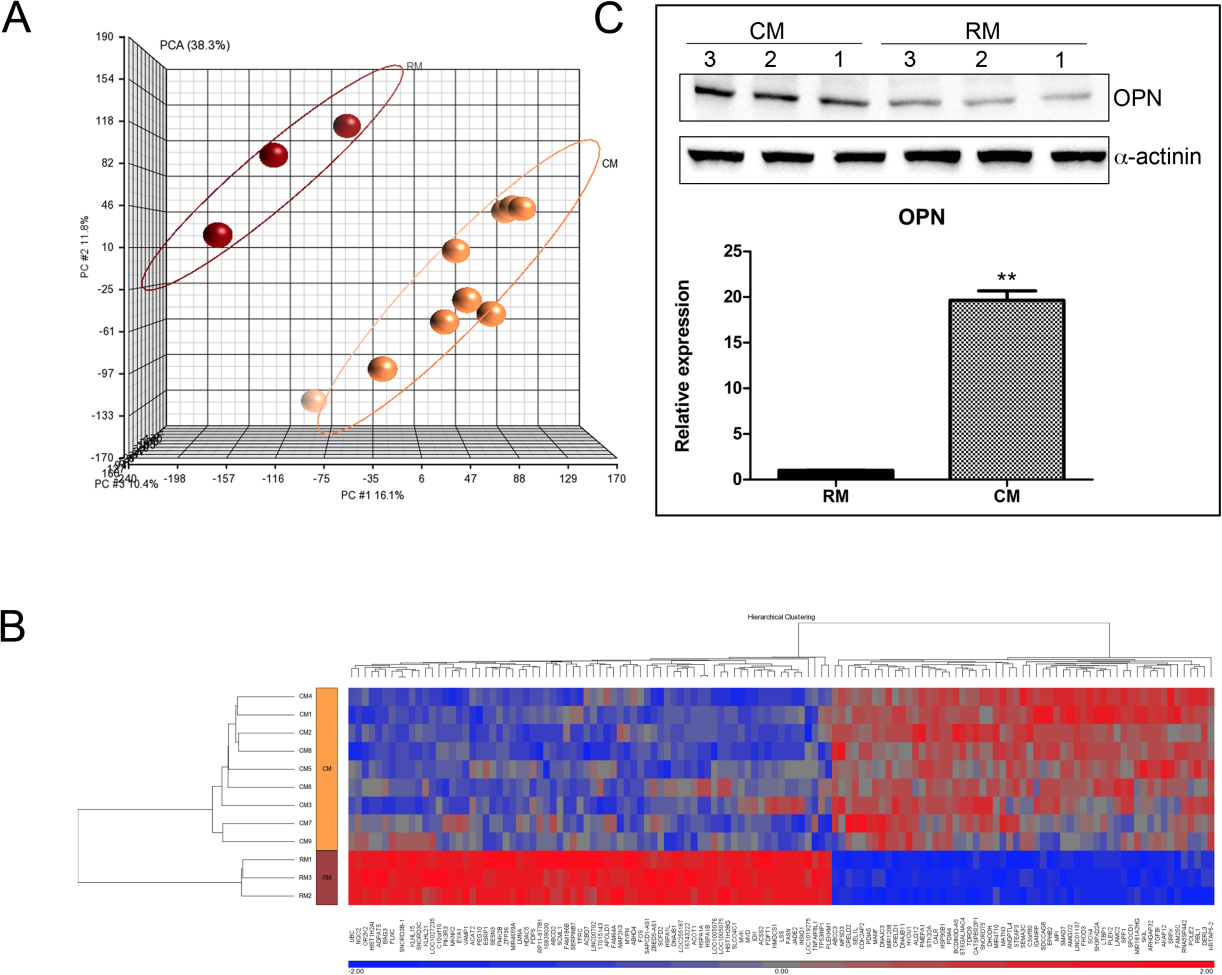
Molecular characterization of omental fat CM-treated PANC-1 cells. (A) PCA of gene expression microarray data. The PCA graphs of global gene expression data were computed using Partek GS, version 6.6; RM control samples are shown as red spheres, n = 3; CM samples are shown as yellow spheres, n = 9. (B) Affymetrix microarray hierarchical clustering performed on mRNA of PANC-1 cells treated with omental fat CM compared to RM. A colored bar indicating the standardized log2 intensities accompanies the expression profile. (C) qRT-PCR validation of the expression levels of OPN in PANC-1 cells pretreated with omental fat CM compared to RM, n = 6. Cells were analyzed by Western blot for the expression of *OPN* protein levels. α-actinin was used as a protein loading control, n = 6.

We next sought to define the gene expression profile of pancreatic cancer cells exposed to omental fat CM using a cDNA microarray analysis. PANC-1 cells were pretreated with omental fat CM or RM for 24 hours. Principal component analysis (PCA) was initially performed to identify and visualize differences between samples. Fig 5A depicts two distinct uncorrelated clusters, the CM-treated and the RM-treated cells. Microarray analysis performed with a stringent statistical significance cutoff of P< .05 (with false discovery rate correction) revealed 129 differentially expressed genes with at least a 1.5-fold change (Fig 5B). Of them, many were previously reported as being associated with tumor proliferation, migration, invasion, and metastasis. They include *ANGPTL4*, *TGFBI*, *AMIGO2*, *OPN*, *SKIL*, *SMAD7*, *LTBP-4*, *SOX4* and *HYOU1*, which were significantly increased in CM-treated PANC-1 cells compared with RM pretreatment (*P*< .05; Table 2). In contrast, *INSIG1*, *SERPINB7*, *HDAC5*, and *TP53INP1* were down-regulated (*P*< .05; Table 2): these genes function as tumor suppressors and are associated with cellular migration, drug resistance and angiogenesis [30–40]. Three of the most up-regulated genes in cells pretreated with omental fat CM were angiopoietin-like 4 (*ANGPTL4*), transforming growth factor beta induced (*TGFBI*) and secreted phosphoprotein 1/osteopontin (*OPN*). Interestingly, both *ANGPTL4* and *OPN* belong to the matricellular proteins family- nonstructural extracellular matrix (ECM)–associated glycoproteins secreted by cancer cells into the extracellular environment [41, 42]. Of them, *OPN*, a recently reported pro-tumorigenic modulator in pancreatic cancer, was one of the most up-regulated genes (a 2.89-fold increase) in CM pretreated PANC-1 cells [32]. In order to validate our mRNA array results, we next used qRT-PCR to examine *OPN* mRNA expression in PANC-1 cells pretreated with omental fat CM. Fig 5C depicts the overexpression of *OPN* mRNA in PANC-1 cells treated with CM compared to treatment with RM. In addition, lysates from PANC-1 cells pretreated with omental fat CM or RM were subjected to Western blot analysis for *OPN* protein expression. Consistent with microarray and qRT-PCR analyses, there was a remarkable difference in *OPN* levels indicating that omental fat induces *OPN* expression both at protein and RNA levels (Fig 5C).

**Table 2:** Cancer-related genes identified by mRNA array analysis of PANC-1 cells pre-treated with omental fat conditioned medium (CM) versus control regular medium (RM).

Gene ID	Description	Fold Change (CM/RM)	*P*-value *t*-test
ANGPTL4	Angiopoietin-like 4	5.474	6.32E-06
SPP1/OPN	Secreted phosphoprotein 1 /Osteopontin	2.892	0.00025
TGFBI	Transforming growth factor, beta-induced	2.529	2.30E-05
AMIGO2	Adhesion molecule with Ig-like domain 2	2.274	0.00020
SOX4	SRY-related high-mobility group box 4	2.23	7.24E-05
SKIL	SKI-like oncogene	1.725	7.45E-05
SMAD7	SMAD family member 7	1.625	8.90E-05
HYOU1	Hypoxia up-regulated 1	1.595	3.31E-06
LTBP-4	Latent transforming growth factor-beta binding protein 4	1.53	0.000448
INSIG1	Insulin induced gene 1	-3.010	0.0007
SERPINB7	Serpin peptidase inhibitor	-2.211	7.24E-05
HDAC5	Histone deacetylase 5	-1.637	6.21E-06
TP53INP1	Tumor protein p53 inducible nuclear protein 1	-1.577	0.000905

These results strongly support our hypothesis that omental fat-secreted factors alter the gene expression profile of pancreatic cancer cells, thus increasing their aggressive biological behavior. Further research is needed to better understand the exact molecular deregulations related to the pancreatic cancer- omental fat crosstalk.

## Discussion

Approximately one-third of pancreatic cancer patients will develop omental metastases, implying that the omentum is a common microenvironment for metastatic PDAC cells. The tumor microenvironment has gradually become recognized as a key contributor to cancer progression and drug resistance [43–45]. However, the omentum has never been studied in that context in pancreatic cancer, and to the best of our knowledge, this study is the first to describe the pro-tumorigenic effects of omental fat on PDAC tumor cells. Two recent studies described the effects of adipocytes and human adipose tissue stem cells on cellular proliferation and invasion in the setting of pancreatic cancer [46, 47]. Ji et al. have shown that adipose tissue-derived stem cells (ADSCs) can promote the proliferation and invasion of pancreatic cancer cells in vitro, whereas White et al. have demonstrated that intratumoral adipocytes promote murine pancreatic cancer growth. In addition, Ksiąźek's group has shown that senescent omentum-derived human peritoneal mesothelial cells (HPMCs) stimulate proliferation, migration and invasion of colorectal and pancreatic cancers *in vitro* and *in vivo* creating a metastatic niche for peritoneal spread. These senescent HPMCs facilitate their peritoneal adhesion via the expression of cell-bound ICAM-1 [48, 49]. Recently they have also shown that under certain conditions HPMCs are capable of inhibiting growth of colorectal and pancreatic cancers in a mechanism involving the anti-adhesive capabilities of soluble ICAM-1 [50]". Unlike those reports, we sought to initially evaluate the omentum as a whole rather than to study its various components. This experimental model enabled us to better recapitulate the actual complex biology involved in PDAC omental metastasis.

The present study results suggest that the omentum has an active role in the process of PDAC metastatic growth and development. Our data demonstrated that the omentum augments different PDAC cellular characteristics, resulting in a more aggressive biological behavior. Omental fat increased both PDAC cellular proliferation and the proportion of cells in the S-phase, indicating that omental fat possibly secretes growth-promoting factors which regulate the G1/S checkpoint. We also demonstrated that omental fat augments migration and invasion; both of which are crucial for cancer metastasis. Taken together, these fat-induced pro-tumorigenic effects may explain how solitary viable cancer cells thrive in the omentum. We assume that these augmented fundamental properties of cancer metastasis enable cellular proliferation, growth, and migration along the omentum, resulting in extensive omental involvement, generally termed “omental cake”.

'Several recent studies have demonstrated the role of the tumor microenvironment in chemoresistance [43, 44]; however, little is known about the specific role of adipose tissue in that context. Our results suggest that omental fat CM may confer PDAC cellular resistance to gemcitabine. They showed that omental fat CM increased the survival rate of gemcitabine-treated PDAC cells. Moreover, it prevented chemotherapy-induced apoptosis, and reduced the percentage of both necrotic and apoptotic cells. Interestingly, our data demonstrated a consistent anti-apoptotic effect of the omental fat CM, unrelated to the evaluated dosage of gemcitabine. In contrast, they showed a prominent protective effect of omental fat on PDAC cell survival, as shown by the XTT assay, which was noted only for the lower doses of the drug. This can be explained by the different biological activities evaluated by XTT and Annexin-PI apoptosis assay. While XTT evaluates proliferation, the Annexin-PI assay measures apoptosis and necrosis. In the XTT assay, when PDAC cells are treated with higher doses of gemcitabine (>1 μM), the protective effect of the omental CM is nullified. It is possible that the CM does not enable the cells to proliferate under such conditions, although its protective effect against cellular death is lasting. As such, the cells survive, but they do not proliferate. These results may support previous observations that adipocytes impair chemotherapy-induced apoptosis in leukemia [51] and ovarian cancer [12]. Several mechanisms could potentially induce chemoresistance by the tumor microenvironment, one of which is the local release of soluble paracrine factors that promote cell survival and tumor growth [52]. We assume that adipokines may similarly affect the chemoresistance of PDAC cells. Two such adipokines are VEGF and IL-6, which were increased in our experiments; both of which were reported to induce cancer cell survival via enhanced resistance to chemotherapy in PDAC [53].

We used two previously described animal models [7, 54] for our in- vivo experiments. Initially, we implanted viable human omental tissue into the flank of nude mice, followed by co-local injection of PDAC cells; this model did not fully represent our in vitro experiments and it posed several technical obstacles. In the second model, cancer cells were pre- treated with omental fat CM for 48 hours and then injected SC. Apparently, the second model has two major advantages: it recapitulates our in vitro methodology and it enabled us to demonstrate that the omentum enhanced an ongoing pro-tumorigenic effect on PDAC cells, independent of continuous exposure. To avoid potential protumorigenic effects induced by the mice innate omentum we preferred a SC rather than an intraperitoneal (IP) implantation model.

As expected, the in vivo data supported our initial in vitro results. We were intrigued by the observation that fat-induced pro-tumorigenic effects occurred with no need for continuous interaction between the fat and the cancer cells. Our results demonstrated that “priming” PDAC cells with omental fat significantly increased cellular proliferation, resulting in increased tumor growth. These data imply that a relatively short exposure of PDAC cells to omental fat-secreted factors may result in cellular re-programming which promotes an aggressive phenotype of the PDAC cancer cells. In line with our results, Dirat et al recently showed that breast cancer cells cocultivated with mature adipocytes exhibited heightened invasive phenotypic behavior [7]. Those authors concluded that adipocytes participate in a highly complex vicious cycle orchestrated by breast cancer cells to promote tumor progression. Interestingly, our in vivo data showed that omental fat also increased angiogenesis. This may be explained by various pro-angiogenic factors secreted by the different cells of the omentum, i.e., VEGF [55].

Collectively, our results strongly support the premise that the omentum has an active role in the formation and growth of pancreatic cancer omental metastasis. To reinforce our results and to expand our findings, we investigated the molecular properties possibly involved in the potential crosstalk between PDAC cells and the omentum. Initially, we characterized the omental fat secretome. Since omental fat and subcutaneous fat are two distinct fat depots [56], it seemed reasonable to select this tissue as our experimental control as did others in their visceral fat investigations [57]. Our LC-MS/MS analysis provided a long list of distinct omental fat proteins. Focusing on potential paracrine PDAC-fat interactions, we selected extracellular soluble molecules that included certain proteins associated with tumor growth, survival, and metastasis (i.e., MMP8, ZA2G, TSP1, FINC, TIMP1, IL-6 and MSLN), as well as a few that were reported in the context of cellular adhesion and chemotaxis (i.e., MSLN, ZA2G, TSP1 and FINC). Of all molecules, MSLN was the most distinctive and probably the most interesting factor. Indeed, omental metastases are frequent in pancreatic cancer, although synchronous involvement of other peritoneal surfaces is not uncommon. Both the peritoneum and the omentum are covered by mesothelial cells which normally secrete MSLN, a 40kD glycosyl-phosphatidylinositol–linked cell surface glycoprotein which is present on the surface of the mesothelial cells and may be involved in cell adhesion [58]. Our LC-MS/MS identified the secreted isoform of MSLN which is capable of binding to CA125/MUC16, a heavily glycosylated membrane-associated protein which is overexpressed on the surface of pancreatic cancer cells [59–62]. Two recent reports have shown that MSLN promotes pancreatic cancer cell motility and invasion [59, 63], while others have demonstrated that it may play an important role in cell adherence [61], cell survival and proliferation, tumor progression [63, 64] and chemoresistance [65–67]. Taken together, these data indicate that it is possible that MSLN has a crucial role in the adhesion of PDAC cells to the peritoneal surfaces and in the development of PDAC carcinomatosis. We therefore believe that MSLN should be further investigated as a potential therapeutic target in PDAC peritoneal spread.

In an effort to understand the molecular mechanisms involved in the phenotypic behavior of PDAC cells following treatment with omental fat CM, we performed a gene expression array. Our analysis demonstrated substantial genetic deregulations; specifically, omental fat- treated PDAC cells overexpressed various tumor associated genes. One of the up-regulated genes was the sex-determining region Y (SRY) box 4 (*SOX4*), a transcription factor which is overexpressed in many types of human cancers. A recent meta-analysis of available gene expression profiling data identified *SOX4* as a member of a consensus gene expression ‘cancer signature’ [68]. Mounting data show that *SOX4* has a critical role in the regulation of epithelial-to-mesenchymal transition-related transcription factors, i.e., ZEB, Twist and Snail [69]. Hasegawa et al. recently reported that *SOX4* overexpression in pancreatic cancer patients was associated with adverse outcomes [70]. Combining those data with our own current results, it seems reasonable to assume that *SOX4* may have a role in the pathogenesis of pancreatic cancer omental metastasis. Interestingly, few of the most up-regulated genes belong to the extracellular matrix proteins, i.e., transforming growth factor, beta-induced (*TGFBI*) and *OPN*. This is a recently emerging subgroup of regulatory proteins which play an important role in many of the classic cancer hallmarks [71]. *TGFBI* expression was significantly increased in peritoneal tumors compared to primary ovarian tumors [72]. As to *OPN*, two recent studies have demonstrated that it significantly increased the invasion and proliferation of PDAC cell lines in vitro and their growth in vivo. Binding of *OPN* to CD44 promotes cell migration through kinase cascades, such as phospholipase Cγ, protein kinase C, phosphatidylinositol 3-kinase (PI3K) and Akt, which also regulates cell cycle progression and growth factor-mediated survival [32, 73]. These data suggest that both *TGFBI* and *OPN* may have a specific role in the development of peritoneal metastasis.

### Conclusions

The findings of our current study highlight the role of the tumor microenvironment in the development and progression of omental metastases. These data suggest a potential crosstalk between omental fat (the soil) and pancreatic cancer cells (the seed). The results imply that the omentum may induce cellular reprogramming in tumor cells which promotes their aggressiveness via different biological functions. These data are novel, particularly in the context of omental spread in pancreatic cancer. We are aware that these data are preliminary and therefore should be validated by a more inclusive study aimed at elucidating the exact cells, molecules and means of delivery involved in cancer- omental fat crosstalk. Understanding the mechanisms essential for this process may be highly relevant to the development of new therapeutic strategies in the management of patients with metastatic pancreatic cancer.
